# Regulatory role of G9a and LSD1 in the Transcription of Olfactory Receptors during Leukaemia Cell Differentiation

**DOI:** 10.1038/srep46182

**Published:** 2017-04-07

**Authors:** Hyeonsoo Jung, Yun-Cheol Chae, Ji-Young Kim, Oh-Seok Jeong, Hoon Kook, Sang-Beom Seo

**Affiliations:** 1 Department of Life Science, College of Natural Sciences, Chung-Ang University, Seoul 156-756, Republic of Korea; 2 Environmental Health Center for Childhood Leukaemia and Cancer, Department of Pediatrics, Chonnam National University Hwasun Hospital, Hwasun 519-809, Republic of Korea

## Abstract

Recent studies have reported the ectopic expression of olfactory receptors (ORs) in non-olfactory tissues, however, their physiological roles were not well elucidated. ORs are expressed in and function in different types of cancers. Here, we identified that the H3K9me2 levels of several *OR* promoters decreased during differentiation in the HL-60, human myeloid leukaemia cell line, by all-*trans*-retinoic acid (ATRA). We found that the differential *OR* promoters H3K9me2 levels were regulated by G9a and LSD1, resulting in the decrease of *OR*s transcription during HL-60 differentiation. G9a and LSD1 could regulate the expression of *OR*s in several non-olfactory cells via the methylation and demethylation of H3K9me2. In addition, we demonstrated that knockdown of *OR* significantly reduced cell proliferation. Therefore, the epigenetic regulation of *OR*s transcription is critical for carcinogenesis.

G9a is a histone methyltransferase (HMTase), belonging to the Su(var)3-9 family of proteins, and it catalyses histone H3K9 mono- and di-methylation associated with transcriptional repression^1^. Transcriptional regulation by G9a and its homologous HMTase G9a-like protein (GLP), is involved in many cellular processes. For example, G9a (or GLP) is critical for early embryo development and embryonic stem cell differentiation in the mouse^2^. G9a knockout mice marked with dramatically reduced H3K9 methylation, exhibited severe growth defects in early development^2^,^3^. G9a also attenuates DNA methylation levels through the suppression of ubiquitin-like with PHD and ring finger domains 1 (UHRF1) which plays an essential function to maintain DNA methylation during HL-60 differentiation^4^,^5^,^6^. In HL-60 cells, G9a represses Janus kinase 2 (JAK2), the catalyst of H3Y41 phosphorylation, resulting in the inhibition of JAK2-H3Y41P-HP1α pathway-mediated leukaemogenesis^7^,^8^. FAD-dependent amine oxidase, lysine-specific demethylase 1 A (LSD1) is a unique protein with the ability to catalyse the demethylation of H3K4me2 and H3K9me2, and therefore act as a transcriptional repressor or activator, respectively^9^,^10^. LSD1 was first reported to demethylate H3K4me2 and repress transcription with the CoREST complex. However, LSD1 also catalyses the demethylation of H3K9me2 with the androgen or oestrogen receptor, and acts as a transcriptional activator^9^,^10^,^11^.

Olfactory receptors (ORs) are G protein-coupled receptors mainly expressed in the olfactory sensory neurons (OSNs) of the olfactory epithelium. ORs play roles in detecting odorants and guiding OSNs axons to the brain^12^,^13^. Mammals typically have ~1,000 OR genes organised in gene clusters on most chromosomes, but about 350 putative functional genes encode ORs in humans^14^. ORs are the largest gene superfamily in mammals, but each olfactory neuron expresses only a single OR allele, according to the “one receptor, one neuron” rule^15^. Recent studies have shown that the singular OR expression in mammals is mediated by the HMTase G9a and demethylase LSD1^16^,^17^. Before a single OR gene is chosen, OR genes are marked with H3K9me2 by G9a which has a critical role in maintaining OR silencing in neurons^17^. Upon further differentiation, all OR genes in a neuron exhibit the hallmarks of constitutive heterochromatin, H3K9me3 and H4K20me3, and are completely silenced^18^. In developmental stage, LSD1 catalyses the demethylation of H3K9me2 of a single OR allele, and that selected OR becomes activated while all other ORs remain repressed. The feedback driven by OR expression induces the unfolded protein response (UPR) and results in the expression of adenylyl cylase 3 (Adcy3), a negative regulator of LSD1^19^. In this way, G9a and LSD1 can regulate single OSN to express and maintain the expression of only one OR^20^.

Recently, it was revealed that different ORs are expressed in non-olfactory tissues including tissues from the testis, tongue, heart, blood, prostate and brain, as well as in spermatozoa^21^,^22^,^23^,^24^,^25^,^26^,^27^. Ectopically expressed ORs have their own physiological functions. For example, hOR17-4 is expressed in testis and spermatozoa and has crucial roles in sperm chemotaxis^22^. OR10J5 is expressed in the heart and is involved in angiogenesis^27^. Additionally, overexpressed OR51E2 (PSGR, prostate-specific G-protein-coupled receptor) enhanced oncogenesis in prostate tissue through activation of NF-κB^28^. However, the physiological roles of ORs in these tissues are still not well understood.

Here, we identified a decrease in OR expressions during human myeloid leukaemia cell line, HL-60, differentiation by all-*trans*-retinoic acid (ATRA). We found that G9a was recruited to the *OR* promoters while the levels of LSD1 at *OR* promoter were reduced, which caused repression of *OR* gene expression. Knockdown of *OR10G2* inhibited the cell proliferation, suggesting that expression of ORs might have regulatory roles in leukaemia cell maintenance.

## Results

### ORs are down-regulated during ATRA-mediated HL-60 differentiation

**Figure 1:**
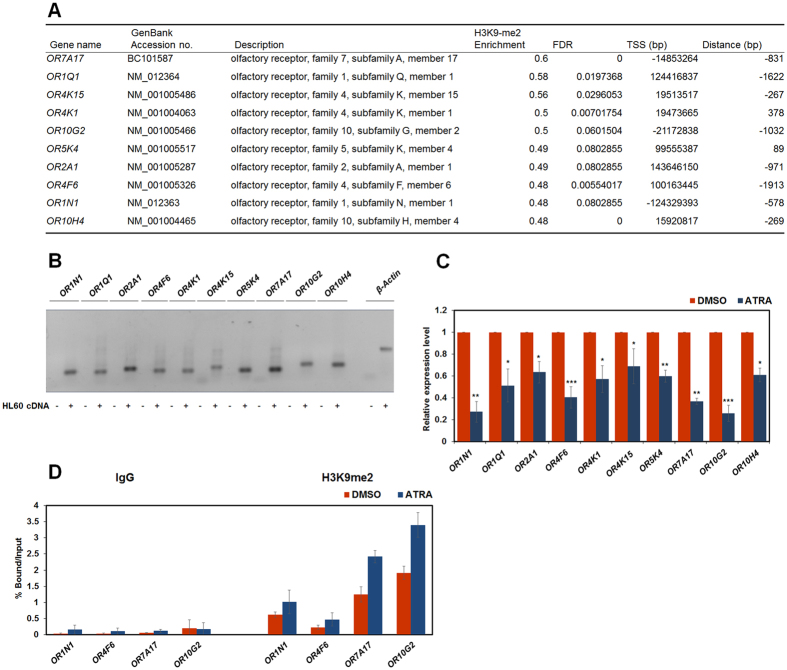
Expression of ORs is repressed during ATRA-induced HL-60 differentiation. HL-60 cells were treated with ATRA (1 μM) or DMSO for 48 h. (**A**) The H3K9me2 level of *OR*s during HL-60 differentiation was analysed from ChIP-chip analysis data of previous research^29^. (**B**) The expression of 10 *OR*s in HL-60 cells was confirmed by RT-PCR using primer pairs for qRT-PCR (Supplementary Table S1). β-actin was used as a positive control. Full-length gel is presented in Supplementary Figure S4. (**C**) The mRNA levels of each *OR* were analysed using qRT-PCR. All results represent at least three independent experiments (±SDs). *P < 0.05, **P < 0.01, ***P < 0.001. (**D**) ChIP analyses were performed using anti-H3K9me2 antibodies, and results were confirmed by qRT-PCR. Recruitment of H3K9me2 to the *OR1N1, OR4F6, OR7A17*, and *OR10G2* promoters was normalised by input.

Previously, it was shown that the H3K9me2 level of *OR51E1* promoter increased during ATRA-mediated differentiation of HL-60 cells^29^. Recently, the function of ORs in non-olfactory tissues has been investigated. It has been reported that the activation of ORs is important for the maintenance of blood progenitor cells in Drosophila^25^. Therefore, we hypothesised that differential H3K9me2 levels of ORs promoter may imply a regulatory role of ORs in leukaemic haematopoiesis. We analysed ChIP-chip data and found that H3K9me2 levels of other ORs were also increased upon ATRA treatment. (Fig. 1A). The differential expressions of the top 10 ORs in ChIP-chip data sets, as determined by changes in the H3K9me2 level, was measured during HL-60 differentiation. First, we checked the expression of these *OR*s in HL-60 cells using RT-PCR (Fig. 1B). Next, HL-60 cells were treated with ATRA for 48 h and the mRNA expression levels of *OR*s were analysed by qRT-PCR. The *OR* expression was down-regulated during the ATRA-mediated differentiation of HL-60 cells (Fig. 1C). We focused on *OR1N1, OR4F6, OR7A17*, and *OR10G2*, all of which showed significant decreases in transcription levels compared to the other OR genes analysed. We conducted ChIP-qPCR to confirm the promoter H3K9 methylation levels of these *OR*s from ChIP-chip data. Consistently, H3K9me2 levels of *OR* promoters increased after 48 h of treatment of ATRA (Fig. 1D). Therefore, we concluded that the expression of *OR*s is repressed during ATRA-meditated differentiation in HL-60 cells through increased levels of promoter H3K9me2.

### G9a and LSD1 regulate expression of ORs in HL-60 cells

**Figure 2:**
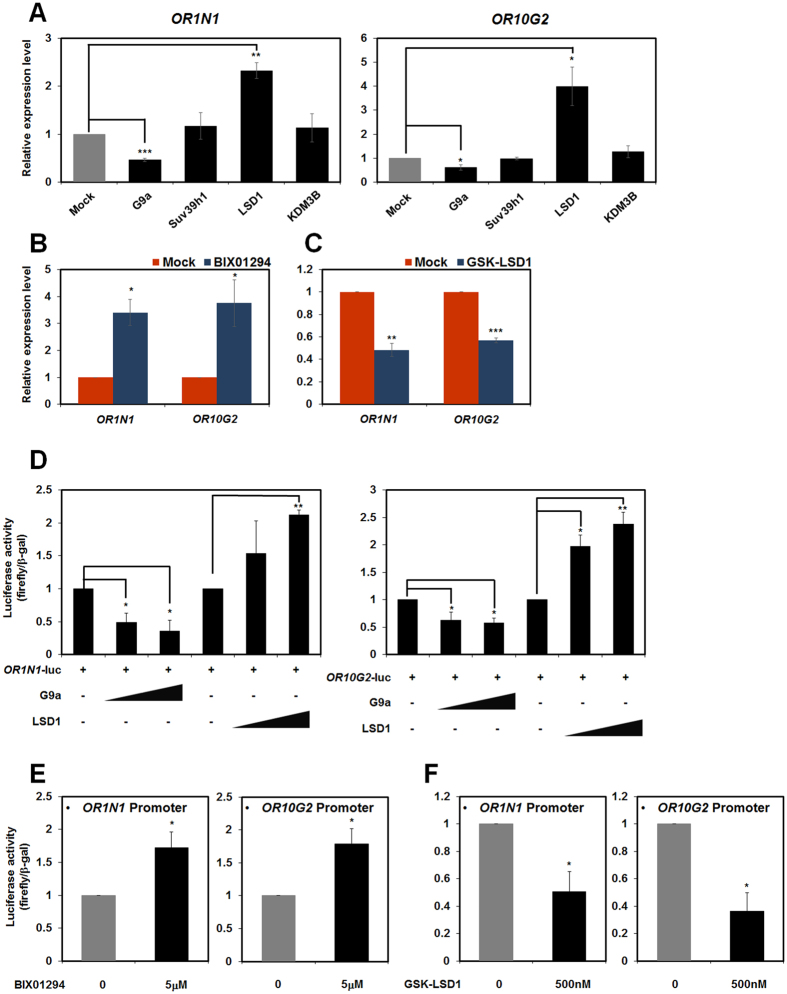
Expression of ORs is regulated by G9a and LSD1. (**A**) HCT116 and H1299 cells were transfected with pEGFP-G9a, pCMV-Suv39h1, pCMV-Flag-LSD1, and pCMV10-Flag-KDM3B. The mRNA levels of *OR1N1* in HCT116 cells and *OR10G2* in H1299 cells were analysed using qRT-PCR. (**B**) HL-60 cells were treated with the G9a inhibitor BIX01294 (5 μM). After 48 h, qRT-PCR was performed to compare the expression levels of each *OR*. (**C**) HL-60 cells were treated with the LSD1 specific inhibitor, GSK-LSD1 (500 nM). After 24 h, qRT-PCR was performed to compare the expression levels of each *OR*s. (**D**) 293T cells were co-transfected with the pEGFP-G9a, pCMV-Flag-LSD1 and pGL4.12-*OR1N1* or pGL4.12-*OR10G2* promoters. Luciferase activities was measured 48 h after transfection. (**E**,**F**) 293T cells were transfected with pGL4.12-*OR1N1* or pGL4.12-*OR10G2* promoters. 24 h after transfection, BIX01294 (5 μM) or GSK-LSD1 (500 nM) were added for 24 h, and luciferase activities were measured. Luciferase activities were normalised to that of β-galactosidase. (**A**–**F**) All results represent at least three independent experiments (±SDs). *P < 0.05, **P < 0.01, ***P < 0.001.

Epigenetic regulations of tumour suppressors, miRNAs, and transcription factors involved in the differentiation of leukaemic cells^8^,^29^,^30^,^31^,^32^,^33^,^34^,^35^. To further investigate the epigenetic regulation of ORs, we overexpressed several epigenetic modifiers that are well known to regulate H3K9me2. H3K9me2 is added by G9a and Suv39h1, and removed by KDM3B^1^,^29^,^36^,^37^. We also tested LSD1, which functions as both a transcriptional activator and repressor by removing H3K4me2 and H3K9me2, respectively^9^,^10^. G9a repressed expression of *OR1N1* and *OR10G2* in HCT116 and H1299 cells in manners similar to those reported in olfactory neurons, while Suv39h1 had no effect (Fig. 2A). In addition, LSD1 activated *OR1N1* and *OR10G2* expression in HCT116 and H1299 cells, suggesting that LSD1 can function as a H3K9me2 demethylase in OR expression (Fig. 2A). However, KDM3B could not regulate the expression of ORs. The regulation of *OR4F6* and *OR7A17* by G9a and LSD1 were also detected in 293T cells (Supplementary Figure S1A). These data indicated that G9a and LSD1 could regulate ORs expression in olfactory neurons and other cell lines including HCT116, H1299 and 293T. Furthermore, the expression levels of *OR1N1, OR4F6, OR7A17*, and *OR10G2* in HL-60 cells increased and decreased following shRNA knockdown of G9a and LSD1, respectively (Supplementary Figure S1B and C). Treatment with the G9a specific inhibitor, BIX01294, also resulted in increased expression of *OR*s (Fig. 2B and Supplementary Figure S1D). However, the GSK-LSD1, LSD1 specific inhibitor, repressed the expression of *OR*s (Fig. 2C and Supplementary Figure S1E). Taken together, these results suggest that G9a represses, and LSD1 activates *OR* expression in HL-60 cells. Moreover, MEF G9a knockout cells exhibited increased expression of the mouse orthologues of *OR1N1* and *OR10G2, Olfr351* and *Olfr1510* compared to wild type cells (Supplementary Figure S1F). These data demonstrate that G9a and LSD1 play key regulatory roles in the expression of ORs in different cell lines.

We tested whether the regulatory roles of G9a and LSD1 affect the promoter activity of ORs using luciferase reporter assay. As expected, G9a could repress *OR1N1* and *OR10G2* promoter activities, while LSD1 activated them (Fig. 2D). Furthermore, consistent with previous data, BIX01294-mediated G9a inhibition resulted in activation of ORs promoter activities and GSK-LSD1 treatment led to repression of *OR*s promoter activities (Fig. 2E and F). We obtained the same results for *OR4F6* and *OR7A17* (Supplementary Figure S2A–C). These results suggest that G9a and LSD1 function as transcriptional regulators of *OR*s through affecting the promoter region.

### The change in OR expression caused by G9a and LSD1 is mediated via the level of H3K9me2

**Figure 3:**
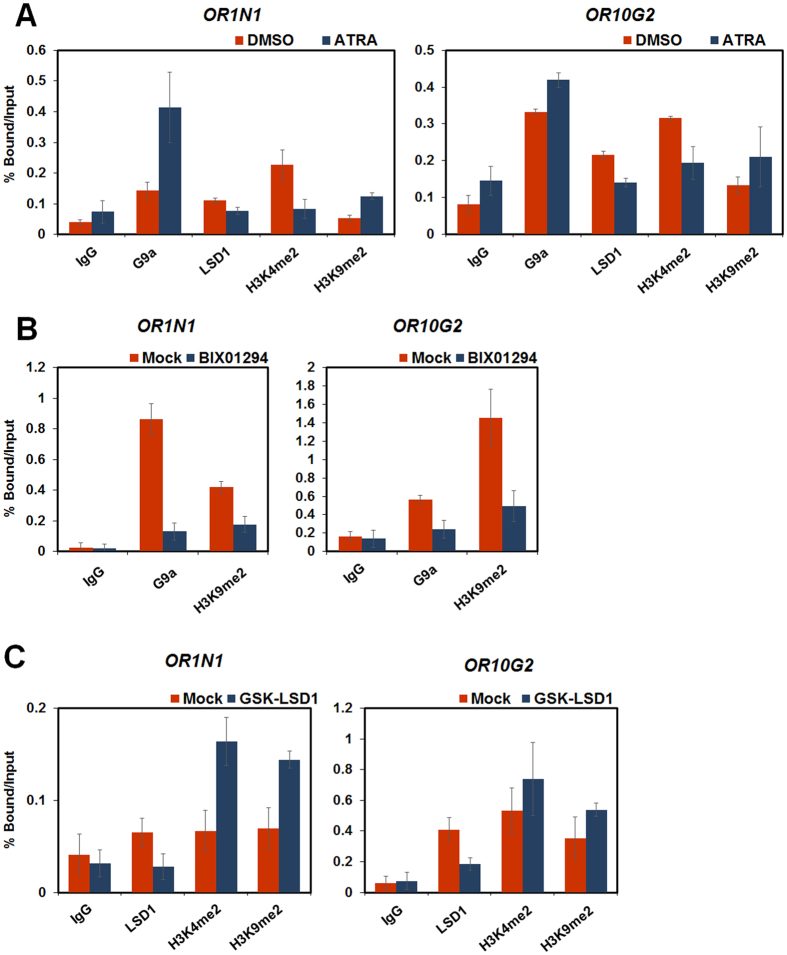
G9a and LSD1 regulate OR expression through the methylation and demethylation of H3K9. (**A**) ChIP analyses of the *OR1N1* and *OR10G2* promoters in ATRA-treated HL-60 cells were conducted using anti-G9a, anti-LSD1, anti-H3K4me2, anti-H3K9me2, and anti-IgG antibodies and were examined via qRT-PCR analyses. (**B**,**C**) HL-60 cells were treated with BIX01294 (5 μM) or GSK-LSD1 (500 nM) for 48 or 24 h, respectively. (**B**) ChIP analyses of the *OR1N1* and *OR10G2* promoters were performed using anti-G9a, anti-H3K9me2, and anti-IgG antibodies and examined by qRT-PCR analyses. (**C**) Using anti-LSD1, anti-H3K4me2, anti-H3K9me2, and anti-IgG antibodies, ChIP analyses were performed. The results were analysed by qRT-PCR. (**A**–**C**) These results are shown as mean ± SDs. (n = 3).

During ATRA-mediated HL-60 differentiation, we showed that H3K9me2 levels on the promoters of each ORs increased (Fig. 1D). We further examined whether G9a or LSD1 regulate OR expression via the methylation or demethylation of H3K9 respectively, by ChIP-qPCR. The recruitment of G9a to *OR* promoters increased 48 h after ATRA treatment, while the amount of LSD1 at *OR* promoters decreased (Fig. 3A and Supplementary Figure 3A). Furthermore, H3K9me2 levels were increased at *OR* promoters. However, during HL-60 differentiation, the level of H3K4me2 at *OR1N1, OR4F6, OR7A17*, and *OR10G2* promoters decreased, consistent with the low *ORs* mRNA expression levels. To further confirm the roles of G9a and LSD1 in HL-60 differentiation, we treated HL-60 cells with BIX01294 and GSK-LSD1, respectively. Treatment with BIX01294 inhibited the recruitment of G9a to *OR* promoter regions and resulted in decreased H3K9me2 levels (Fig. 3B and Supplementary Figure 3B). In contrast, treatment with GSK-LSD1 resulted in decreased LSD1 recruitment to the *OR* promoters and inhibited the demethylation of H3K9me2 (Fig. 3C and Supplementary Figure 3C). Unexpectedly, GSK-LSD1 treatment also increased H3K4me2 level, suggesting that LSD1 might demethylate H3K4me2 and H3K9me2 on *OR* promoter regions. Taken together, these data suggest that G9a and LSD1 regulate H3K9 methylation levels during ATRA-mediated HL-60 differentiation.

### Knockdown of *OR10G2* inhibited the cell proliferation and induced HL-60 differentiation

**Figure 4:**
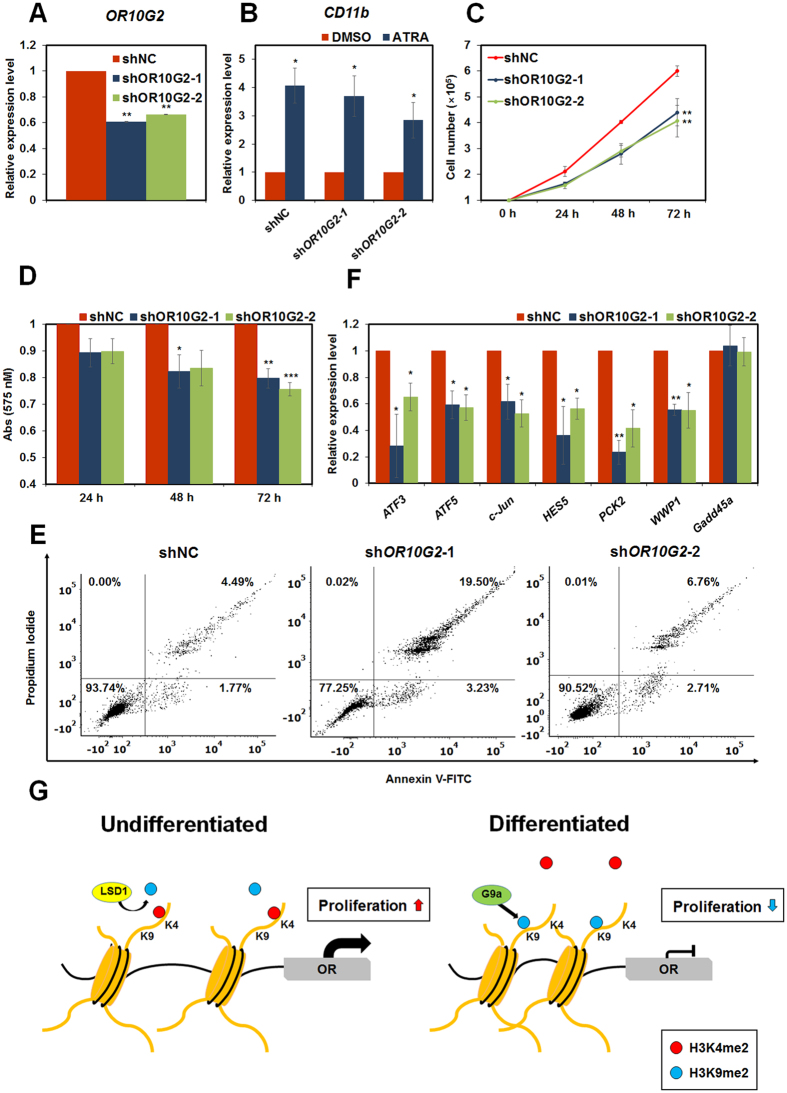
Down-regulation of OR10G2 decreases cell proliferation. (**A**) The mRNA level of *OR10G2* in stable *OR10G2* knockdown cells was measured by qRT-PCR analyses. (**B**) Stable *OR10G2* knockdown HL-60 cells were treated with ATRA (1 μM) or DMSO for 48 h. qRT-PCR was performed to compare the expression levels of *CD11b*. (**C**) Cell counting assays were performed using stable *OR10G2* knockdown HL-60 cells. (**D**) Cell proliferation was assessed through MTT assay in which stable *OR10G2* knockdown HL-60 cells were used. (**A**–**D**) Results are expressed as means ± SDs. (n = 3). *P < 0.05, **P < 0.01, ***P < 0.001. (**E**) Apoptosis was measured in control and stable sh*OR10G2* HL-60 cell lines by PI and Annexin V-FTIC staining. Cells were stained with Annexin V-FITC and PI for 2 h and 30 min, respectively, and analysed by FACS. (**F**) The mRNA levels of proliferation-related genes were measured by qRT-PCR. The results represent at least three independent experiments (±SDs). *P < 0.05, **P < 0.01. (**G**) A model of the regulation of ORs transcription by G9a and LSD1 during HL-60 differentiation is presented.

Given the fact that the expression of ORs decreased during ATRA-mediated HL-60 differentiation, ORs may play important roles in leukaemia cell proliferation or differentiation. To analyse the function of ORs in HL-60 cells, we designed shRNAs targeting *OR10G2* and generated stable *OR10G2* knockdown HL-60 cells (Fig. 4A). We first analysed the expression of HL-60 differentiation marker, CD11b^38^. *OR10G2* knockdown had no effect on *CD11b* expression level (Fig. 4B). Next, we performed cell counting and MTT assays whether cell proliferation changes caused by *OR10G2* knockdown (Fig. 4C and D). Importantly, the growth of stable *OR10G2* knockdown HL-60 cells was significantly lower than that of control HL-60 cells. Using fluorescence-activated cell sorting (FACS) analysis, we found that sh*OR10G2*-1 and sh*OR10G2*-2 cells exhibited decreased proportions of live cells (93.74% to 77.25% and 90.52%, respectively) and increased proportions of apoptotic cells (4.49% to 19.50% and 6.72%) compared to control cells (Fig. 4E). These data suggest that OR10G2 affects the cell proliferation in HL-60 cells. To further analyse molecular mechanism by *OR10G2* knockdown, we checked the expression level of several cell proliferation-related genes, *ATF3, ATF5, C-Jun, HES5, PCK2* and *WWP1* and anti-proliferation gene, *Gadd45a,* as a control in stable *OR10G2* knockdown HL-60 cells^39^,^40^,^41^,^42^,^43^,^44^,^45^. As expected, these genes are significantly downregulated in stable *OR10G2* knockdown HL-60 cells, while the mRNA level of *Gadd45a* was not changed (Fig. 4F). These data show the regulatory roles of ORs in the proliferation of HL-60 cells, although ORs do not regulate directly HL-60 differentiation. Collectively, our results demonstrate that the regulation of *OR* transcription by G9a and LSD1 plays an important role in the proliferation of HL-60 cells, and that aberration of this regulation may contribute to leukaemogenesis.

## DISCUSSION

Recent studies have revealed the functions of ectopically expressed ORs in various types of cells. ORs have functions in sperm chemotaxis, angiogenesis, maintenance of blood progenitor cells, and oncogenesis^22^,^25^,^28^,^46^. Moreover, ORs are also expressed in germinal cells, embryos, and developing heart and muscle, suggesting that they function during developmental stages^47^,^48^,^49^,^50^,^51^,^52^. ORs in non-olfactory tissues do not appear to be related to olfaction activity but rather perform their own different tissue-dependent functions^53^.

Here, we demonstrated the epigenetic regulation of ORs and functions of OR10G2 during ATRA-mediated HL-60 differentiation. Transcription of *OR*s was activated by LSD1 and repressed by G9a in different cell types including HL-60 cells (Fig. 2 and Supplementary Figure S1 and S2). When HL-60 cells were differentiated via ATRA treatment, G9a was directly recruited to the *OR* promoters and the recruitment of LSD1 was reduced, catalysing the di-methylation of H3K9 and resulting in decreased OR expression (Fig. 3 and Supplementary Figure S3). Stable *OR10G2* knockdown HL-60 cells exhibited decreased cell proliferation rate and increased apoptosis by reducing proliferation-related genes, suggesting that the function of OR10G2 in HL-60 cells may influence the survival of leukaemic cells (Fig. 4C–E). In summary, LSD1 activates the expression of ORs by demethylation of H3K9me2 in HL-60 cells. During ATRA-mediated HL-60 differentiation, G9a was recruited to the *OR* promoters and repressed the expression of *OR*s by mediating the methylation of H3K9. This in turn, caused the reduced cell proliferation of HL-60 cells (Fig. 4G).

In our previous ChIP-chip data, more than 30 *OR*s were regulated during HL-60 differentiation^29^. In this story, we analysed the epigenetic regulation of 4 OR genes, OR1N1, OR4F6, OR7A17 and OR10G2, and focused on the function of OR10G2 in HL-60 cells. However, there is a possibility that other ORs are involved in various cellular processes including proliferation via epigenetic regulations.

LSD1 has been known as both of H3K4 and H3K9 demethylase. We found that LSD1 activated *OR* genes expression in HL-60 cells (Fig. 2 and Supplementary Figure S1 and S2). Interestingly, LSD1 inhibition resulted in the increase of the bivalent transcriptional histone markers, H3K4me2 and H3K9me2 (Fig. 3C and Supplementary Figure S3C). It is possible that the changes in H3K4me2 level is a secondary effect of LSD1 inhibition. Also, LSD1 is known as demethylase of H3K4me1/2 and H3K9me2, not H3K4me3^10^,^11^,^37^,^54^,^55^. Therefore, in this study, even though LSD1 demethylates H3K4me2, H3K4me3 might remain on *OR* gene promoter, and reduced H3K9me2 level result in the activation of *OR* expression. To further delineate the demethylase activities of LSD1 on *OR* promoters, further studies are needed.

Despite our current data demonstrating an oncogenic function for ORs, several studies have suggested that ORs may act as tumour suppressors^24^,^56^,^57^,^58^. The activation of OR2AT4 and OR51B5 in myelogenous leukaemia K562 cells, decreased proliferation and enhanced apoptosis and differentiation^56^,^57^. In addition, activation of OR51E1 in prostate cancer cells suppressed cell proliferation^24^,^58^. However, stimulation of ORs was also reported to promote cell invasiveness and metastasis^59^. Interestingly, OR51E2 has been reported to function as an oncogene and tumour suppressor in prostate cancer^24^,^28^. The activation of OR51E2 was reported to inhibit the proliferation of prostate cancer cells, while overexpression promoted the tumour development. These contradictions may be compatible with a hypothesis that a single OR can bind with multiple ligands, which have different potentials for downstream signal activation^28^. According to data from Olfaction DB, a single ligand can activate several ORs^60^. Even though ORs are receptors, the expression and activation by chemical agonists of ORs may lead to different physiological results. In cells, GPCRs can be activated by several molecules including hormones, peptides and other proteins^61^. Previous studies used chemical agonists to activate ORs, but it is possible that they also activate other ORs or GPCRs. As a result, the ORs in human leukaemic cells can be activated by molecules present in the human environment and may have their own pathways that differ from the activation by chemical ligands. It can be expected that differentially repressed ORs might lose their roles in cell proliferation. Since the ligand that activates OR10G2 has not yet been identified, the effect of OR10G2 activation in HL-60 cells remains unanswered. Nevertheless, OR10G2 is overexpressed in leukaemia cells and functions in cell proliferation in our study. Therefore, it is tempting to speculate that regulation of OR transcription can be an attractive new therapeutic target in leukaemogenesis.

## Methods

### Plasmid constructs

For the luciferase assay, genomic DNA was prepared and the *OR1N1, OR4F6, OR7A17 and OR10G2* promoter region (−1487 to 0, −1500 to +20, −1365 to 0 and −1022 to 0, respectively) was inserted into the pGL4.12-basic vector (Promega). The *OR1N1, OR4F6, OR7A17 and OR10G2* promoter sequence were amplified from human genomic DNA using primer pairs (Supplementary Table S1). pEGFP-G9a, pCMV-Flag-LSD1, pCMV-Suv39h1, and pCMV10-Flag-KDM3B were previously described^6^,^8^,^62^. Short hairpin RNAs (shRNAs) against G9a were previously described and against LSD1 and OR10G2 were designed using the siRNA sequence designer software (Clontech)^6^. A double-stranded oligonucleotide for shRNA plasmid construction was produced using primers from the 5′ to the 3′ end (Supplementary Table S1). These oligonucleotides were inserted into the AgeI/EcoRI site of the pLKO.1 TRC vector (Addgene).

### Cell culture

HCT116, H1299 and HL-60 cells were grown in RPMI 1640, and G9a^−/−^ MEF and 293 T cells were cultured in Dulbecco’s modified Eagle’s medium supplemented with 10% heat-inactivated FBS and 0.05% penicillin-streptomycin at 37 °C in a 5% CO_2_ atmosphere. For differentiation, HL-60 cells were seeded in 60 mm plate with 5 × 10^6^ per ml and treated with 1uM ATRA or DMSO (Sigma). After 48 h incubation, the cells were harvested and used for each experiments. For inhibition of G9a and LSD1, HL-60 cells were seeded in 60 mm plate with 5 × 10^6^ numbers and treated with the 5 μM BIX01294 and 500 nM GSK-LSD1, respectively. After 48 h and 24 h incubation respectively, cells were harvested and used in experiments.

### Antibodies

Antibodies specific for β-Actin (sc-47778), LSD1 (sc-271720; Santa Cruz Biotechnology), G9a (07-551), H3K9me2 (07-441), H3K4me2 (07-030), and mouse IgG (12-371; Millipore) were purchased.

### RNA interference

To produce virus particles, 293T cells were co-transfected with plasmids encoding VSV-G, NL-BH, and the shRNAs against G9a, LSD1 and OR10G2. Two days after transfection, the soups containing the viruses were collected and used to infect HL-60 cells in the presence of polybrene (8 μg/ml).

### Reverse Transcription PCR and qRT-PCR

Total RNA was isolated from cells using Tri-RNA Reagent (FAVORGEN). After cDNA synthesis, the cDNA was quantified and then subjected to analysis of mRNA expression. The PCR primers used are presented in Supplementary Table S1. Dissociation curves were examined after each PCR run to ensure amplification of a single product of the appropriate length. The mean threshold cycle (C_T_) and standard error values were calculated from individual C_T_ values obtained from triplicate reactions per stage. The normalised mean C_T_ value was estimated as ΔC_T_ by subtracting the mean C_T_ of β-actin. The value ΔΔC_T_ was calculated as the difference between the control ΔC_T_ and the values obtained for each sample. The n-fold change in gene expression, relative to an untreated control, was calculated as 2^−ΔΔCT^.

### Luciferase assay

For the luciferase assay, 293T cells were seeded in 48-well plates and co-transfected with the indicated expression plasmid and the pGL4.12-*OR1N1,* pGL4.12-*OR4F6,* pGL4.12-*OR7A17* and pGL4.12-*OR10G2* reporter plasmid using polyethylenimine. After 48 h, the cells were harvested and subjected to a luciferase assay (Promega). The level of β-galactosidase activities was used to normalise the reporter luciferase. Data are expressed as the means of four replicates from a single assay. All results shown are representative of at least three independent experiments.

### ChIP and real-time PCR analysis

Cells were harvested and subsequently cross-linked with 1% formaldehyde. Briefly, 1% formaldehyde was added to the medium for 10 min at room temperature, followed by the addition of 125 mM glycine for 5 min at room temperature. HL-60 cells were centrifuged, and the resulting pellets were washed once with 1X PBS. The cell pellets were resuspended in SDS lysis buffer (1% SDS, 10 mM EDTA, 50 mM Tris-HCl [pH 8.1]). Cells were then sonicated, and the lysates were subjected to immunoprecipitation using the indicated antibodies. The immunoprecipitates were eluted and reverse cross-linked, after which the DNA fragments were purified for PCR amplification. The DNA fragments were then purified and PCR amplified for quantification using each PCR primer pair (Supplementary Table S1). The thermal cycler conditions were as follows: 3 min of holding at 95°C followed by 45 cycles at 95 °C for 10 s, 56 °C for 10 s, and 72 °C for 30 s (Bio-Rad). The mean threshold cycle (C_T_) and standard error values were calculated from individual C_T_ values, obtained from duplicate reactions per stage.

### MTT (3-(4,5-dimethylthiazol-2-yl)-2,5-diphenyltetrazolium bromide) Assay

shNC and sh*OR10G2* HL-60 cells were seeded in 48-well plates with 5 × 10^5^ per ml per each well. After 24, 48, and 72 h, MTT was added to the cells (final concentration 0.5 mg/ml), after which they were incubated further for 4 h at 37 °C. DMSO was added (200 μl), and The OD was determined using a spectrophotometer at the wavelength of 575 nm.

### FACS analysis

To measure the effect of OR10G2 on apoptosis, HL-60 sh*OR10G2* stable cells were washed. Immediately before flow cytometric analysis, the cells were treated with RNase A (20 mg/ml) and stained with Annexin V-FITC (Biobud) for 2 h and propidium iodide (SIGMA) for 30 min. HL-60 cells were then subjected to FACS analysis using a BD FACSAria^TM^ II (BD bioscience), and the data were analysed using FCS Express 6 Plus (De Novo Software).

### Statistical analysis

Data are expressed as means ± SDs of three or more independent experiments. Statistical significance (*P* < 0.05) was calculated using functions in Microsoft Excel. Differences between groups were evaluated by one-way analysis of variance (ANOVA), followed by a student’s t-test or Bonferroni test, as appropriate.

## Additional Information

**How to cite this article**: Jung, H. *et al*. Regulatory role of G9a and LSD1 in the Transcription of Olfactory Receptors during Leukaemia Cell Differentiation. *Sci. Rep.* **7**, 46182; doi: 10.1038/srep46182 (2017).

**Publisher's note:** Springer Nature remains neutral with regard to jurisdictional claims in published maps and institutional affiliations.

## Supplementary Material

Supplementary Information
