# Author Correction: Regulatory role of G9a and LSD1 in the Transcription of Olfactory Receptors during Leukaemia Cell Differentiation

**DOI:** 10.1038/s41598-018-35591-1

**Published:** 2018-11-19

**Authors:** Hyeonsoo Jung, Yun-Cheol Chae, Ji-Young Kim, Oh-Seok Jeong, Hoon Kook, Sang-Beom Seo

**Affiliations:** 1 Department of Life Science, College of Natural Sciences, Chung-Ang University, Seoul, 156-756 Republic of Korea; 2 Environmental Health Center for Childhood Leukaemia and Cancer, Department of Pediatrics, Chonnam National University Hwasun Hospital, Hwasun, 519-809 Republic of Korea

Correction to: *Scientific Reports* 10.1038/srep46182, published online 07 April 2017

This Article contains an error in the Abstract, where:

“Here, we identified that the H3K9me2 levels of several OR promoters decreased during differentiation in the HL-60, human myeloid leukaemia cell line, by all-trans-retinoic acid (ATRA)”.

should read:

“Here, we identified that the H3K9me2 levels of several OR promoters increased during differentiation in the HL-60, human myeloid leukaemia cell line, by all-trans-retinoic acid (ATRA)”.

