# Supplementary material for: Regulatory role of G9a and LSD1 in the Transcription of Olfactory Receptors during Leukaemia Cell Differentiation
Source: Sci Rep. 2017 Apr 7;7:46182. doi: 10.1038/srep46182 (PMC5384044; doi:10.1038/srep46182)
Supplement: Supplementary Information [file srep46182-s1.pdf]

Supplemental Information

Regulatory role of G9a and LSD1 in the Transcription of Olfactory Receptors during Leukaemia Cell Differentiation

Hyeonsoo Jung[1], Yun-Cheol Chae[1], Ji-Young Kim[1], Oh-Seok Jeong[1], Hoon Kook[2] and Sang-Beom Seo[1,*]

[1]Department of Life Science, College of Natural Sciences, Chung-Ang University, Seoul 156-756, Republic of Korea

[2]Environmental Health Center for Childhood Leukaemia and Cancer, Department of Pediatrics, Chonnam National University Hwasun Hospital, Hwasun 519-809, Republic of Korea

***Correspondence:** sangbs@cau.ac.kr

Name	Sequence (5'- to -3')	Purpose
pOR1N1 (-1487 to 0) F	CCGCTCGAGGACAGGTCTGAGGTAGGG	Promoter-luc
pOR1N1 (-1487 to 0) R	GAAGATCTGACTCTGCAGCATCTGGA	
pOR4F6 (-1500 to +20) F	GGGGTACCGTGGTGTCCTGAGCCCTT	Promoter-luc
pOR4F6 (-1500 to +20) R	CCGCTCGAGTGCCTCTGTCGGCTGTGA	
pOR7A17 (-1365 to 0) F	GGGGTACCAGCTACAGTGTGCTGCGT	Promoter-luc
pOR7A17 (-1365 to 0) R	CCGCTCGAGCTTTTTTTTTCTATTTAT	
pOR10G2 (-1022 to 0) F	CCGCTCGAGCCTGGAGCAAAAACAGGC	Promoter-luc
pOR10G2 (-1022 to 0) R	GAAGATCTGTCTTTTTGTAGTCTGCT	
OR1N1 (-414 to -527) proximal F	GATGCCAATGACAAAAACTCAA	ChIP
OR1N1 (-414 to -527) proximal R	GAGTGAAGGGCATATGAAGGAC	
OR4F6 (-197 to -329) proximal F	GCTGAGTGAAGAATGTGGAGTG	ChIP
OR4F6 (-197 to -329) proximal R	TTGTCACCAGAAAATACGTTGC	
OR7A17 (-361 to -556) proximal F	AGAGTGTCATGCCTCCCAAC	ChIP
OR7A17 (-361 to -556) proxima R	GCAGAGCAGTGTGGAACAAA	
OR10G2 (-567 to -676) proximal F	CAGGGACCAGATTTACACACAA	ChIP
OR10G2 (-567 to -676) proxima R	ATCCCTTGACTGACATCACCTC	
OR1N1 F	CCAATATCGTTGCCCTGACT	qRT-PCR
OR1N1 R	GCATAAAAAGGGGACGATGA	
OR4F6 F	TGAAGCCAATCACTCTGTGG	qRT-PCR
OR4F6 R	GGGACTGTAAACGAGGGTCA	
OR7A17 F	AATCAGGTCATCCACCTTGC	qRT-PCR
OR7A17 R	TCCCCTGAGCTGATGAGATT	
OR10G2 F	TCCTCAGTCACCGTTCCTCT	qRT-PCR
OR10G2 R	AACCTCCCATTCATGAGCAC	
Olfr351 F	GGTATTTGCCTTAGGGGGTA	qRT-PCR
Olfr351 R	GGGCATAAGTAGGCACTGAA	
Olfr1510 F	TGTGCTCATGAATGGAAAATTA	qRT-PCR
Olfr1510R	CTGCAGGAATGTCACAGATAAA	
LSD1 F	CCCAAAGAAACTGTGGTG	qRT-PCR
LSD1 R	TATGTTCTCCCGCAAAGA	
ATF3 F	GTTAGGATTCAGGCAGCAGT	qRT-PCR
ATF3 R	GTGACACAAACATCGGAAGAG	
ATF5 F	AGTACGTCAAGGACCTGCT	qRT-PCR
ATF5 R	GTGCTGGGATTACAAGCATAAG	
C-Jun F	CAGGTGGCACAGCTTAAACA	qRT-PCR
C-Jun R	TTTTTCTCTCCGTCGCAACT	
HES5 F	ACCGCATCAACAGCAGCATT	qRT-PCR
HES5 R	AGGCTTTGCTGTGCTTCAGGT	
PCK2 F	CTTGGATGAGGTTTGACAGTG	qRT-PCR
PCK2 R	TCACAGTAACACCAGGTGG	
WWP1 F	CTTCACATGACCTGAAGTTGG	qRT-PCR
WWP1 R	AGCCACTGAAGAGGAACAAC	
Gadd45a F	CTGAACCAAATTGCACTGAA	qRT-PCR
Gadd45a R	TCTTTCCATCTGCAAAGTCA	
CD11b F	CGATTCCGTGTTCACCCTGC	qRT-PCR
CD11b R	TGCCGCTTGAAGAAGCCGAG	
shLSD1 3' UTR sense	CCGGGGAGCTCCTGATTTGACAAAGCTCGAGCTTTGTCAAATCAGGAGCTCCTTTTTG	shRNA
shLSD1 3' UTR antisense	AATTCAAAAACCTCGAGGACTAAACTGTTTCGAGCTCGAAACAGTTTAGTCCTCGAGG	
shLSD1 CDS sense	CCGGCCACGAGTCAAACCTTTATTTCTCGAGAAATAAAGGTTTGACTCGTGGTTTTTG	shRNA
shLSD1 CDS antisense	AATTCAAAAAGGTGCTCAGTTTGGAAATAAAGAGCTCTTTATTTCCAAACTGAGCACC	
shOR10G2 CDS #1 sense	CCGGCCCAATCAGGTGGATTACTTTCTCGAGAAAGTAATCCACCTGATTGGGTTTTTG	shRNA
shOR10G2 CDS #1 antisense	AATTCAAAAACCCAATCAGGTGGATTACTTTCTCGAGAAAGTAATCCACCTGATTGGG	
shOR10G2 CDS #2 sense	CCGGCATCATTTACATCCTCACTCACTCGAGTGAGTGAGGATGTAAATGATGTTTTTG	shRNA
shOR10G2 CDS #2 antisense	AATTCAAAAACATCATTTACATCCTCACTCACTCGAGTGAGTGAGGATGTAAATGATG	

Table S1. Primer pair in this study

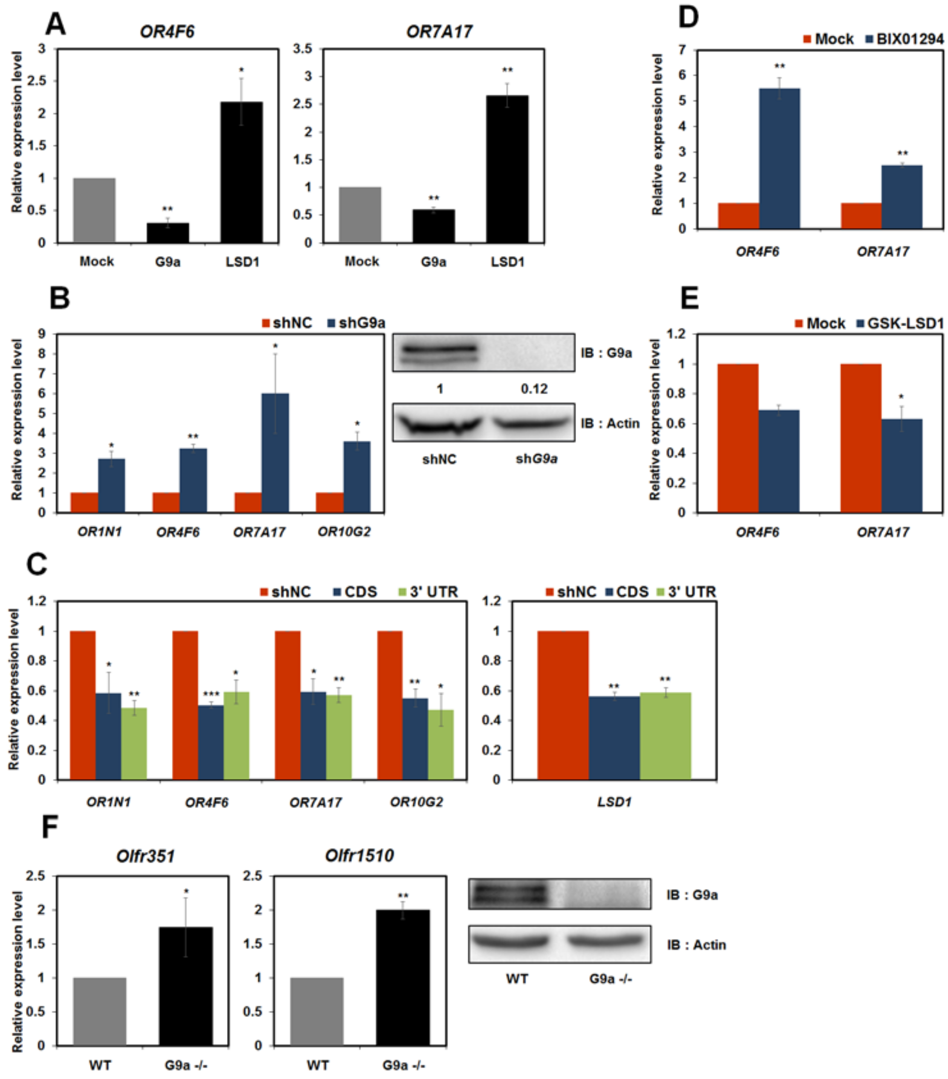

Figure S1. The expressions of *OR4F6* and *OR7A17* is regulated by G9a and LSD1. (A) 293T cells were transfected with transfected with pEGFP-G9a, pCMV-Suv39h1, pCMV-Flag-LSD1 and pCMV10-Flag-KDM3B. qRT-PCR was used to examine the mRNA levels of *OR4F6* and *OR7A17*. **(B-C)** HL-60 cells were treated with sh*G9a* and sh*LSD1* lentivirus, respectively. Each *OR*s expression levels were confirmed by qRT-PCR. **(B)** Knockdown of G9a was confirmed by immunoblot with anti-G9a antibody. β-actin was used as loading control. The G9a level relative to β-actin was analysed by Image J. **(C)** The mRNA levels of *LSD1* in sh*LSD1* were measured by qRT-PCR using primers presented in Supplementary Table S1. **(D-E)** HL-60 cells were treated with G9a inhibitor BIX01294 (5 µM) for 48 h and

LSD1 inhibitor GSK-LSD1 (500 nM) for 24 h, respectively. qRT-PCR was performed to compare the expression levels of *OR4F6* and *OR7A17*. **(F)** *Olfr351* and *Olfr1510* expression levels in G9a knockout MEF cells were analysed using qRT-PCR. G9a level in G9a knockout MEF cells was detected by immunoblot with anti-G9a antibody. β-actin was used as loading control. **(A-G)** All results represent at least three independent experiments (±SDs). * $P < 0.05$, ** $P < 0.01$, *** $P < 0.001$. Full-length blots are presented in Supplementary Figure S4.

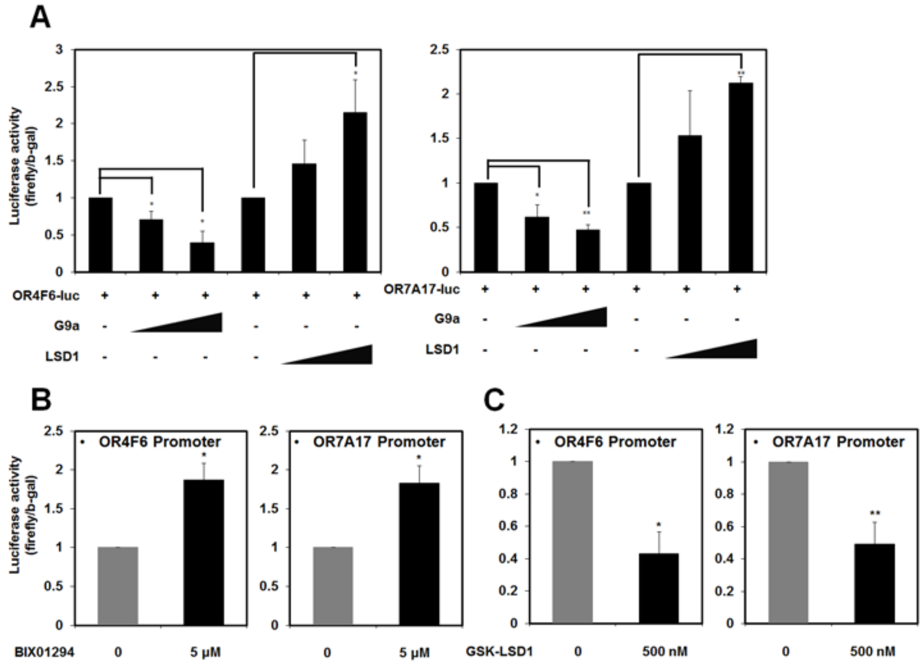

Figure S2. G9a and LSD1 regulates the transcription of *OR4F6* and *OR7A17*. (A) 293T cells were co-transfected with the pEGFP-G9a, pCMV-Flag-LSD1 and pGL4.12-*OR4F6* or pGL4.12-*OR7A17* promoter. Luciferase activities were measured 48 h after transfection. **(B-C)** 293T cells were transfected with pGL4.12-*OR4F6* or pGL4.12-*OR7A17* promoter. 24 h after transfection, BIX01294 (5 μM) or GSK-LSD1 (500 nM) were added for 24 h, and luciferase activities were subsequently measured. **(A-C)** Luciferase activities were normalised to that of β-galactosidase, and the results are presented as means ±SD (n = 3). * $P < 0.05$, ** $P < 0.01$.

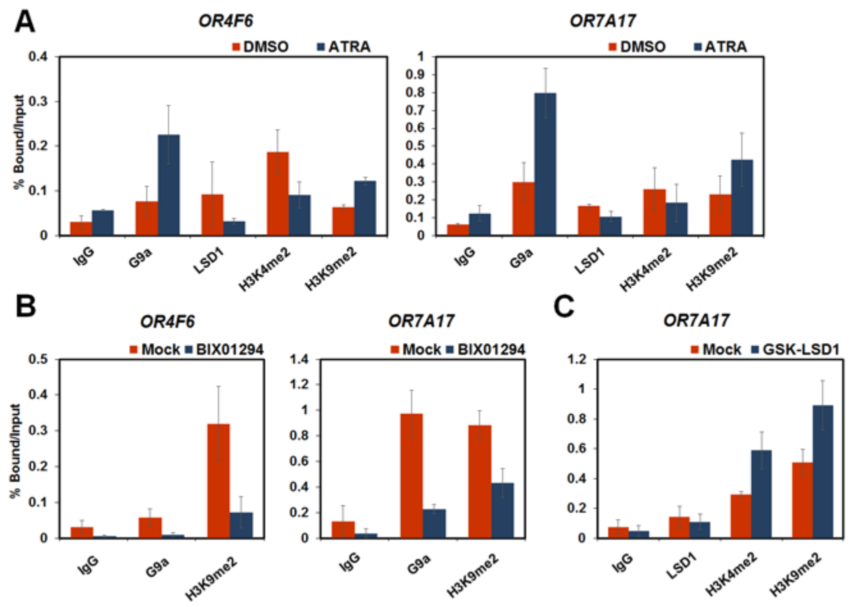

Figure S3. G9a and LSD1 regulate OR4F6 and OR7A17 expression by modulating the methylation and demethylation of H3K9 during HL-60 differentiation. (A) ChIP analyses of the *OR4F6* and *OR7A17* promoters during ATRA-mediated HL-60 differentiation were conducted using anti-G9a, anti-LSD1, anti-H3K4me2, anti-H3K9me2 and anti-IgG and were examined via qRT-PCR analyses. **(B-C)** HL-60 cells were treated with BIX01294 (5 μM) or GSK-LSD1 (500 nM) for 48 or 24h, respectively. **(B)** ChIP analyses of the *OR4F6* and *OR7A17* promoter were performed using anti-G9a, anti-H3K9me2 and anti-IgG and examined by qRT-PCR analyses. **(C)** Using anti-LSD1, anti-H3K4me2, anti-H3K9me2 and anti-IgG, ChIP analysis of the *OR7A17* promoter was performed. The results were analysed by qRT-PCR. **(A-C)** All results represent at least three independent experiments (±SDs).

Figure 1B

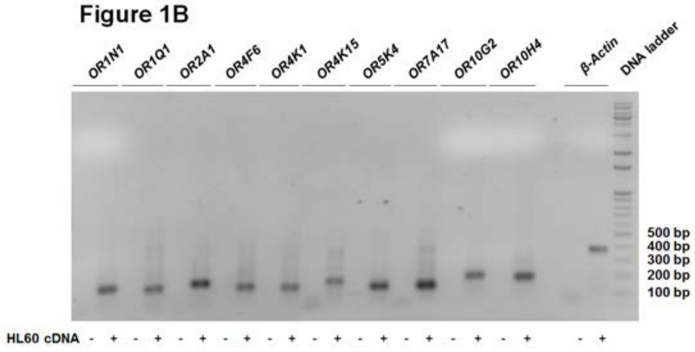

Supplementary Figure S1B

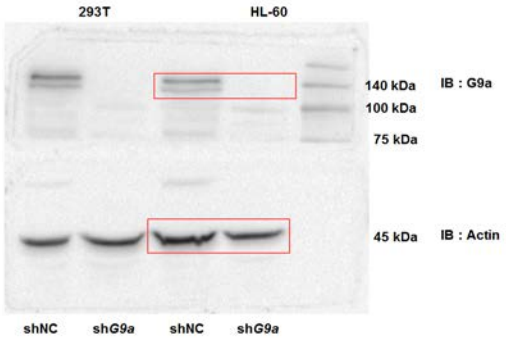

Supplementary Figure S1F

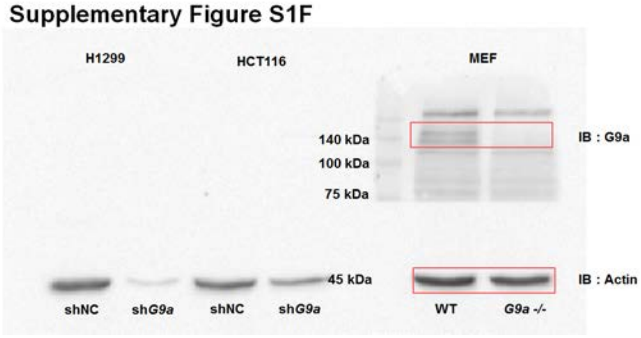

Figure S4
